# Humeral metastasis from a sacrococcygeal chordoma: a case report

**DOI:** 10.1186/1752-1947-5-339

**Published:** 2011-08-01

**Authors:** Negar Azarpira, Said Solooki, Sepideh Sepidbakht, Ramin Mardani

**Affiliations:** 1 Department of Pathology, Shiraz University of Medical Sciences, Shiraz, Iran; 2 Department of Orthopedics, Shiraz University of Medical Sciences, Shiraz, Iran; 3 Department of Radiology, Shiraz University of Medical Sciences, Shiraz, Iran

## Abstract

**Introduction:**

Chordomas are rare tumors of the skeletal system that arise from an intra-osseous benign precursor of notochordal cells. They are mainly locally aggressive. However, metastases to other sites, including the humeri, resulting in pathological fractures have been reported. We report the case of a patient with a metastatic chordoma that produced a pathologic fracture of the humerus.

**Case presentation:**

We report the case of a 60-year-old Iranian woman who presented with a fracture of her right humerus following a minor trauma. She had a history of a sacrococcygeal chordoma. Histological and immunohistochemical studies of the fracture site suggested the diagnosis of a chordoma.

**Conclusions:**

Chordoma is a rare tumor and rarely metastasizes, but it should be considered in the differential diagnosis of epithelioid bone tumors. The only current effective treatment for this type of tumor is carbon ion therapy. There is currently no effective medical therapy available for advanced chordoma, and this type of tumor is not very responsive to radiotherapy.

## Introduction

Chordomas are rare, low-grade, primary malignant bone tumors arising from primitive notochord remnants of the axial skeleton. The sacrum is the most common anatomical site of origin, accounting for 50% to 60% of all cases, followed by the skull base region (spheno-occipital and/or nasal), accounting for 25% to 35% of cases; the cervical vertebrae, accounting for approximately 10% of cases; and the thoracolumbar vertebrae, accounting for approximately 5% of cases [1-4].

These tumors are mainly locally aggressive, with a high incidence of local recurrence and a poor long-term prognosis. Metastatic lesions have been reported in the liver, lungs, lymph nodes, peritoneum, skin, heart, humeri, brain, and distant regions of the spine [1-7].

## Case presentation

**Figure 1:**
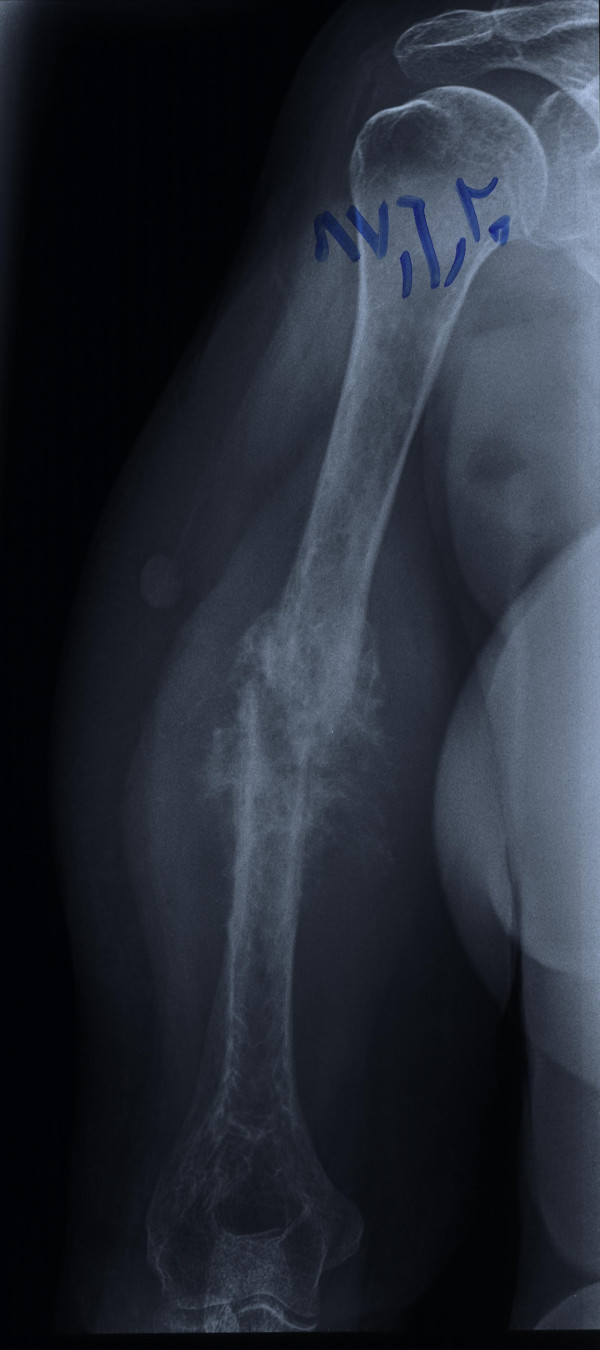
**Plain radiograph showing a tumor characterized by a fracture in the mid-shaft of the right humerus with a large soft tissue mass**.

**Figure 2:**
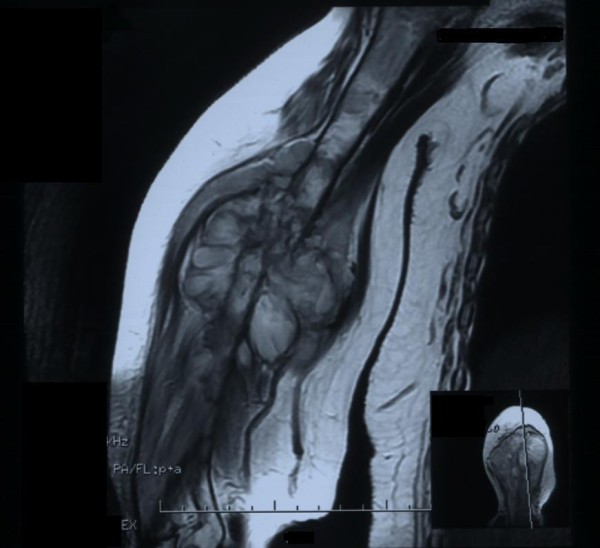
**Magnetic resonance imaging scan showing extensive soft tissue mass with bone destruction of the left humerus**.

**Figure 3:**
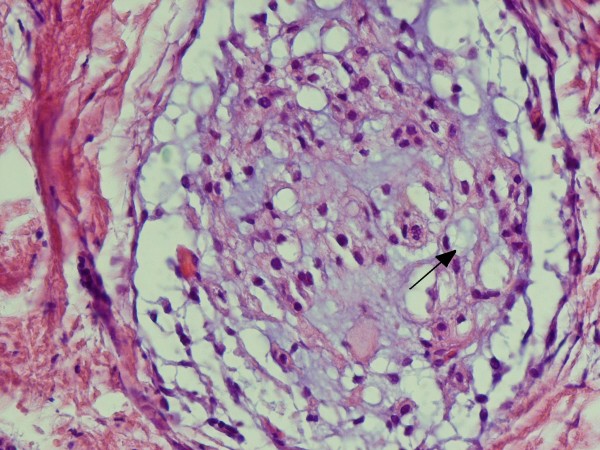
**Typical lobulated appearance of a chordoma with small nests of tumor cells on a pale blue myxoid background**. Prominent intra-cytoplasmic vacuolization can be seen (arrow) (hematoxylin and eosin stain, ×100).

**Figure 4:**
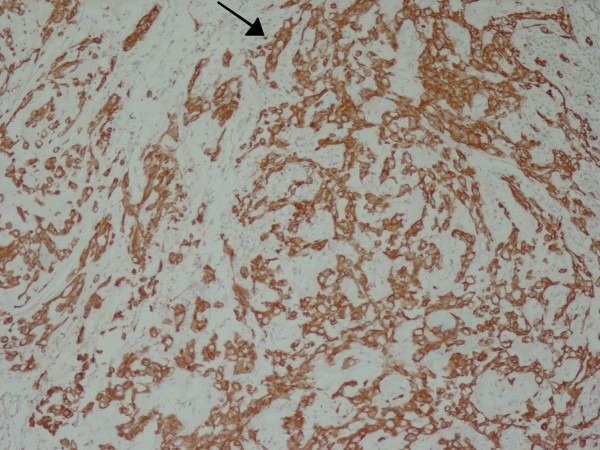
**The tumor cells show strong reactivity with antibodies to ethidium monoazide (arrow) (original magnification, ×100)**.

A 60-year-old Iranian woman presented to our hospital with pain and swelling in her right arm following a minor fall. Our initial physical examination revealed her to be in a good general condition. She was normotensive, with a pulse rate of 92 beats/minute. Her other laboratory values were as follows: body temperature 37°C, hemoglobin 12.2 g/dL, leukocytes 22 cells/mm^3^, platelets 200 U/L, sodium 141 mEq/L, potassium 4 mEq/L, glucose 11.6 mmol/L, blood urea nitrogen 15.1 mmol/L, and creatinine 250 mmol/L. A plain X-ray showed a pathologic fracture in the mid-shaft of her right humerus and a large, ill-defined soft tissue mass (Figure 1). Magnetic resonance imaging (MRI) showed a huge lobulated mass destroying the shaft of the humerus and displacing the surrounding soft tissue. The mass was bright on serial T2-weighted MRI scans and dark on serial T1-weighted MRI scans, with marked enhancement after a gadolinium injection (Figure 2). Four years ago she had been treated for a sacrococcygeal chordoma with surgical resection and post-operative radiotherapy. A local tumor recurrence developed after one year and required additional surgical procedures. A histological examination of the biopsy from the humeral lesion showed a lobulated mass composed of cuboidal to polygonal cells with eccentric nuclei, clear to eosinophilic cytoplasm, and pools of extracellular mucin (arrow in Figure 3). Typically, few tumor cells have a "physaliferous" (bubble-bearing) appearance. On the basis of these findings, we considered differential diagnoses such as myxoid chondrosarcoma, myxoid liposarcoma, primary mixed tumor of the bone, metastasizing pleomorphic adenoma of the salivary gland, metastatic renal cell carcinoma (RCC), or metastatic carcinoma with intra-cytoplasmic mucin from the colon or lung [8,9]. Most metastatic carcinomas have more of the cytological features of malignancy than chordomas do. The tumor cells stained positive for AE1/3 (Figure 4), ethidium monoazide (EMA), S100, and vimentin. Although both chordomas and chondrosarcomas stain with S100 protein, myxoid chondrosarcomas do not stain with keratin and EMA. Additional immunochemistry was performed for CD10, the results of which were negative in this case and usually expressed in RCC. Abdominal and pelvic ultrasonography and computed tomography (CT) of her chest and abdomen were normal. On the basis of this evidence and our review of the original histology following her previous chordoma excision (which was histologically identical to the humeral lesion), a diagnosis of metastatic chordoma was confirmed. She refused any active treatment.

## Discussion

Chordomas arise from the remnant of the fetal notochord and grow slowly. The most common location is the sacrococcygeal region. This tumor is generally reported in adults; most patients are in the fifth to seventh decades of life [10-12]. Classic radiological findings for chordoma are a bony lytic lesion with an accompanying soft tissue mass. The sacrococcygeal lesions are known to metastasize more frequently than the other types, and radiation therapy raises the risk of metastasis. Despite all the available modalities of treatment, the prognosis for this condition remains poor because of the difficulty in gaining surgical clearance and the tumor's propensity to metastasize [11]. Histologically, mitoses and anaplasia can be present in chordomas without adversely affecting the duration of a patient's survival. Previous studies have found that many of the metastatic lesions were asymptomatic or were found during post-mortem examination, which suggests that metastases from chordomas could be considered indolent in these cases [4,5]. Our patient had an asymptomatic rib lesion as well as a large humeral metastasis with pathologic fracture. A pathological analysis of the metastatic tumor revealed features identical to the primary tumor. Metastasis to the long bones is a rare phenomenon.

The biological behavior of chordomas differs from patient to patient. Some patients harbor tumors capable of aggressive behavior both locally and distantly, while others have tumors that behave more indolently. There is no good method for predicting the behavior of individual chordomas [4,11]. Therefore, it seems that aggressive surgical resection with local adjuvant treatment, such as carbon ion therapy, must be undertaken to prevent the development of recurrent lesions and metastatic neoplasms.

## Conclusions

Chordomas are rare tumors. They are relatively radioresistant, and currently there is no effective medical therapy for treating them. Complete local excision is the most effective treatment for achieving a long-term cure. Although these tumors rarely metastasize, they need to be considered in the differential diagnosis of metastatic epithelioid bone tumors.

## Consent

Written informed consent was obtained from the patient for publication of this case report and any accompanying images. A copy of the written consent is available for review by the Editor-in-Chief of this journal.

## Competing interests

The authors declare that they have no competing interests.

## Authors' contributions

NA participated in the histology-related issues and drafted the manuscript. SSo contributed to all of the surgical aspects of patient care and revised the respective sections in the manuscript. SSe participated in the radiological aspects of the case and provided input to the case discussion. RM contributed to the review of the literature and provided clinical insights. All authors read and approved the final manuscript.
